# Human CD72 splicing isoform responsible for resistance to systemic lupus erythematosus regulates serum immunoglobulin level and is localized in endoplasmic reticulum

**DOI:** 10.1186/1471-2172-13-72

**Published:** 2012-12-26

**Authors:** Yuki Hitomi, Takahiro Adachi, Naoyuki Tsuchiya, Zen-Ichiro Honda, Katsushi Tokunaga, Takeshi Tsubata

**Affiliations:** 1 Laboratory of Immunology, School of Biomedical Science, Tokyo Medical and Dental University, 1-5-45 Yushima, Bunkyo-ku, Tokyo, 113-8510, Japan; 2 Department of Immunology, Medical Research Institute, Tokyo Medical and Dental University, 1-5-45, Yushima, Bunkyo-ku, Tokyo, 113-8510, Japan; 3 Department of Human Genetics, Graduate School of Medicine, The University of Tokyo, 7-3-1, Hongo, Bunkyo-ku, Tokyo, 113-0033, Japan; 4 Department of Allergy and Rheumatology, Graduate School of Medicine, The University of Tokyo, 7-3-1, Hongo, Bunkyo-ku, Tokyo, 113-0033, Japan; 5 Doctoral Program in Life System Medical Sciences, Graduate School of Comprehensive Human Sciences, University of Tsukuba, 1-1-1, Tennodai, Tsukuba, 305-8575, Japan; 6 Core Research for Evolutional Science and Technology, Japan Science and Technology Agency, 4-1-8, Honcho, Kawaguchi, 332-0012, Japan

**Keywords:** Polymorphism, Exon skipping, C-type lectin domain

## Abstract

**Background:**

CD72 is an inhibitory co-receptor expressed on B cells. We previously demonstrated significant association of the polymorphism of the *CD72* gene with susceptibility to human systemic lupus erythematosus (SLE) in individuals carrying a SLE-susceptible *FCGR2B* genotype (*FCGR2B-232Thr/Thr*). The human *CD72* locus generates a splicing isoform that lacks exon 8 (CD72Δex8) as well as full-length CD72 (CD72fl), and the *CD72* polymorphism regulates exon 8 skipping.

**Results:**

Here we demonstrated that individuals carrying the disease-protective *CD72* genotype exhibit significantly lower serum immunoglobulin levels than do individuals carrying other *CD72* genotypes (*P* < 0.05). Although expression level of CD72fl in the peripheral blood B cells was similar regardless of *CD72* genotype, the protein level of CD72Δex8 was increased in individuals carrying the disease-protective *CD72* genotype, suggesting a crucial role of CD72Δex8 in regulation of antibody production. By expressing these human CD72 isoforms in mouse cell lines, we further demonstrated that CD72Δex8 is accumulated in endoplasmic reticulum (ER) and fails to regulate BCR signaling whereas human CD72fl is efficiently transported to the cell surface and inhibits signaling through the B cell antigen receptor (BCR), as is the case for mouse CD72.

**Conclusion:**

Human *CD72* polymorphism appears to regulate antibody production as well as susceptibility to SLE by regulating expression of ER-localizing CD72Δex8.

## Background

CD72, a 45 kDa type II membrane protein expressed on B cells, is an inhibitory co-receptor that regulates signaling through the B cell antigen receptor (BCR) [1-6]. Both human and mouse CD72 contains a C-type lectin-like domain in the extracellular region and an immunoreceptor tyrosine-based inhibition motif (ITIM) in the cytoplamic region [1-3]. Mouse CD72 negatively regulates BCR signaling by recruiting Src homology 2 domain-containing protein tyrosine phosphatase-1 (SHP-1) at ITIM [4-6]. However, the signaling function of human CD72 remains unknown.

Four human *CD72* polymorphisms have been identified in the upstream regulatory region and introns [7]. These *CD72* polymorphisms constitute two major haplotypes, CD72*1 and CD72*2. We previously demonstrated that *FCGR2B-Ile232Thr*, a gene polymorphism of FcγRIIb, is significantly associated with SLE in Asian populations, and is a risk factor for SLE only in individuals with CD72*1/1[7,8]. This finding indicates that CD72*2 confers resistance to SLE in individuals carrying *FCGR2B-Ile232Thr.* Two polymorphisms in intron 8 regulate generation of an alternative splicing isoform (CD72Δex8) that skips exon 8 independently; probably act in combination as cis-acting intronic splicing enhancer (ISE) or silencer (ISS) [7]. Exon 8 encodes the C-terminal part of the C-type lectin-like domain and the stop codon, and skipping of it results in replacement of the C-terminal part of the C-type lectin-like domain by a sequence encoded in exon 9 in CD72Δex8. The ratio of mRNA level of CD72Δex8 to that of full-length CD72 (CD72fl) is strikingly higher in B cells from individuals with the CD72*2/2 or CD72*1/2 genotype than in those with CD72*1/1[7]. Because there are no substitutions in exons among different *CD72* haplotypes, these findings strongly suggest that increased CD72Δex8 level, decreased CD72fl level, or both are responsible for the resistance of CD72*2-carrying individuals to SLE.

In the present study, we addressed the functional properties of CD72 isoforms. Analysis of healthy individuals revealed that those with CD72*1 express a significantly lower level of the CD72Δex8 protein in B cells, and show the higher level of serum immunoglobulins than those carrying CD72*2, suggesting that CD72Δex8 regulates the immunoglobulin level as well susceptibility to SLE. Analysis using B cell transfectants expressing CD72Δex8 demonstrated that CD72Δex8 does not regulate BCR signaling but accumulates in the endoplasmic reticulum (ER). Thus, the *CD72* polymorphism regulates antibody production and autoimmunity by modulating the level of ER-localizing CD72Δex8.

## Methods

### Plasmids

cDNAs including the entire coding region of human CD72fl or CD72Δex8 but not nucleotides for the stop codon were obtained by RT-PCR from peripheral blood mononuclear cells (PBMCs) with a pair of specific primers (5′-GCA GAG CTG CTC AGG ACC AT-3′ and 5′-ACC CCA TTC TAC CAT GGG AA-3′). The cDNAs encoding CD72fl and CD72Δex8 were inserted with a pair of oligonucleotides encoding FLAG-tag into the retrovirus expression vector pMX-ires-GFP, and the resulting plasmids were termed pMX-CD72fl and pMX-CD72Δex8, respectively.

The retrovirus expression plasmids pMX-CD72flYF and pMX-CD72Δex8YF encoding the mutants of CD72fl and CD72Δex8, in which tyrosine^7^ is replaced by phenylalanine, were generated by PCR-based site-directed mutagenesis using a specific primer set (5′- GCA GAT CTG AGG TTT GTG AA -3′ and 5′- AAA GGT GAT GGC CTC AGC CA -3′).

### Cells

The mouse B cell lines WEHI-231 and K46μv and the human B cell line Raji were described previously [9,10], and cultured in RPMI 1640 medium supplemented with 10% FCS, 50 μM 2-ME, and 1 mM glutamine. The mouse fibroblast cell line Balb/c-3T3 was cultured in DMEM medium supplemented with 10% FCS and 1 mM glutamine. Retrovirus-mediated gene transfer was performed as described previously [9].

PBMCs were obtained from unrelated healthy Japanese living in the central part of Japan where genetic background is shown to be relatively homogeneous [11]. Informed consents were obtained from these indiciduals prior to collecting samples. Peripheral B lymphocytes were isolated from PBMCs by an autoMACS cell sorter (Miltenyi Biotec, Auburn, CA) using the B cell isolation kit II. This study was approved by the Research Ethics Committees of the Graduate School of Medicine, The University of Tokyo.

### Genotyping

Human *CD72* haplotype was determined by genotyping *CD72*-VNTR, the tag polymorphism, by PCR-SSLP method as described previously [7]. *FCGR2B-Ile232Thr* was genotyped by nested PCR and fluorescence resonance energy transfer (FRET) technology as described previously [12].

### Serum IgG level

Serum IgG levels in healthy individuals were measured by turbidimetric immunoassay.

### Flow cytometry

Peripheral blood B cells were incubated with FITC-labeled anti-human CD72 mAb J4-117 (BD Biosciences, San Jose, CA). B cell transfectants were incubated with rabbit anti-FLAG Ab (Cell Signaling Technology, Danvers, MA), followed by reaction with PE-labeled goat anti-rabbit IgG (Southern Biotech, Birmingham, AL). Alternatively, transfectants were stained with NP-conjugated PE. Cells were then analyzed by flow cytometry using a FACSCalibur (BD Biosciences).

### Generation of anti-CD72Δex8 antibody

Rabbits were immunized with the peptide specific for CD72Δex8 (Ala-Asp-Pro-His-Leu-Thr-Leu), and serum IgG was purified from rabbit serum by ammonium sulfate precipitation. The specificity of the antibody was confirmed by Western blotting of total cell lysates of COS-7 CD72Δex8 transfectant using pre-immune and immune serum.

### Immunoprecipitation and Western blotting

Cells were stimulated with either 0.2 μg/ml NP_15_-coupled BSA (NP-BSA) or 10 μg/ml anti-IgM Ab, and were lysed in Triton X-100 lysis buffer [9,10]. Lysates were immunoprecipitated with anti-FLAG Ab (Sigma-Aldrich, St. Louis, MO) together with protein G-Sepharose (GE Healthcare Life Sciences, Piscataway, NJ). Total cell lysates or immunoprecipitates were separated on SDS-PAGE and were transferred to polyvinylidene difluoride membranes. Membranes were incubated with anti-phosphotyrosine mAb 4G10 (Millipore), anti-β-tubulin mAb TUB2.1 (Seikagaku Kogyo, Tokyo, Japan), rabbit anti-FLAG Ab, rabbit anti-CD72 Ab (Santa Cruz Biotechnology, Santa Cruz, CA), rabbit anti-phospho ERK Ab (Cell Signaling Technology), rabbit anti-SHP-1 Ab (Santa Cruz Biotechnology), or rabbit anti-CD72Δex8 Ab. Proteins were visualized with an ECL system.

### Measurement of intracellular calcium mobilization

Cells were incubated in culture medium containing 5 μg/ml fluo-4-AM (Molecular Probes) for 30 min. Cells were stimulated with 0.2 μg/ml NP-BSA, and were analyzed by flow cytometry using a FACSCalibur.

### Assay of apoptotic cells

Cells were incubated with thapsigargin or staurosporine for 24 h and 48 h, and cells containing hypodiploid DNA were measured by flow cytometry [10].

### Immunocytochemistry

Cells were fixed in 4% PFA for 15 min, washed with PBS, and permeabilized with 0.1% Triton X-100 for 15 min. Cells were then washed with PBS, blocked with 2% BSA for 30 min, and stained for FLAG-tagged human CD72 and organelle-specific marker proteins in ER, mitochondria, Golgi apparatus, early endosomes, and late endosomes using rabbit anti-FLAG Ab, anti-KDEL mAb 10C3 (Stressgen, MI), anti-cytochrome C mAb 6H2.B4 (BD Biosciences), anti-GM130 mAb 35 (BD Biosciences), anti-transferrin receptor mAb H68.4 (Invitrogen-Life Technologies), and anti-lamp1 mAb 1D4B (Southern Biotech), respectively. Cells were then analyzed by laser scanning confocal microscopy (Model LSM 510; Carl Zeiss Inc., Jena, Germany).

### Statistical analysis

The serum IgG level among *CD72* genotypes was analyzed using regression analysis and Kruskal-Wallis rank sum test. Relative expression of CD72Δex8 protein product between *CD72* genotype was analyzed using Mann–Whitney U test. Otherwise, Student’s t-test was used for statistical analysis.

## Results

### Decreased serum immunoglobulin levels in CD72*2-carrying individuals

**Figure 1:**
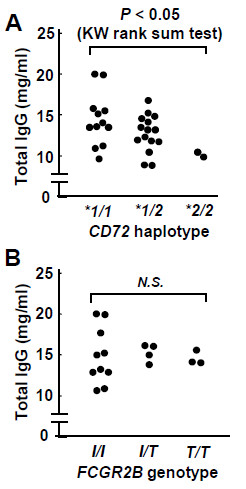
**Serum IgG levels of healthy individuals.** The concentrations of serum IgG were measured by turbidimetric immunoassay. *CD72 IVS8-VNTR* which was the tag polymorphism for human *CD72* haplotypes was genotyped by PCR–simple sequence length polymorphism (SSLP) method, and *FCGR2B-Ile232Thr* was genotyped by nested PCR and fluorescence resonance energy transfer (FRET) technology. The concentrations of serum IgG were compared among *CD72* genotypes (**A**) or *FCGR2B* genotypes (**B**). The serum IgG levels were compared among individuals with CD72*1/1, those with CD72*1/2 and those with CD72*2/2 by Kruskal-Wallis rank sum test, and P-value indicates the estimated probability of rejecting the null hypothesis that rank sum of the serum IgG levels among different *CD72* haplotypes are not different; *P* < 0.05. Additionally, concordance to the model in which the serum IgG levels are in proportion to the number of CD72*1 haplotype was examined by regression analysis, and P-value indicates the estimated probability of rejecting the null hypothesis that serum IgG levels were not in proportion to the number of CD72*1 haplotype; *P* < 0.01.

To address whether the *CD72* polymorphism regulates humoral immunity, we measured the serum IgG levels in 30 healthy volunteers. We genotyped *CD72* IVS8-VNTR which was the tag polymorphism for human *CD72* haplotypes by PCR–simple sequence length polymorphism (SSLP) method [7], and there were the total of 12 with CD72*1/1, the total of 16 with CD72*1/2, and the total of 2 with CD72*2/2. The serum IgG level inversely correlated to the number of CD72*2 allele with statistical significance (Figure 1A, regression analysis: P<0.01; Kruskal-Wallis rank sum test: P<0.05). Additionally, we genotyped *FCGR2B-Ile232Thr* by nested PCR and fluorescence resonance energy transfer (FRET) technology in the same volunteers [12]. There was however no significant difference in the serum IgG level among *FCGR2B-Ile232Thr* genotypes (Figure 1B). Thus, *CD72* polymorphism appears to play a role in the regulation of antibody production.

### Human CD72fl but not CD72Δex8 regulates BCR signaling

**Figure 2:**
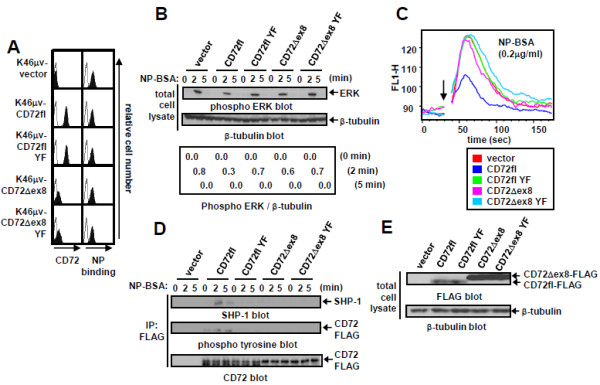
**CD72fl but not CD72Δex8 is efficiently expressed on B cell surface and regulates BCR signaling. A**: Cell surface expression of NP-reactive BCR and human CD72 in K46μv transfectants. Cell surface expression was analyzed by flow cytometry using a FACSCalibur. **B**: ERK phosphorylation induced by antigen NP_15_-coupled BSA (NP-BSA) stimulation in K46μv transfectants. Cells were stimulated with 0.2 μg/ml NP-BSA. Cells were lysed, and total cell lysates were subjected to Western blot analysis. The same blots were reprobed with anti-β-tubulin Ab to ensure equal loading. **C**: Ca^2+^ influx induced by NP-BSA stimulation in K46μv transfectants. Transfectants were loaded with fluo-4-AM, and intracellular free Ca^2+^ was measured with a FACSCalibur. Transfectants were stimulated with 0.2 μg/ml NP-BSA at 30s (indicated by arrow), and measurement of free Ca^2+^ was continued for 180 s. **D**: Phosphorylation and SHP-1 recruitment of CD72 induced by NP-BSA stimulation in K46μv transfectants. Cells were lysed, and CD72 was immunoprecipitated with anti-FLAG Ab. Immunoprecipitates (IP) were subjected to Western blot analysis. The same blots were reprobed with anti-CD72 Ab to ensure equal loading. **E**: Expression of CD72 in total cell lysates in K46μv transfectants. Cells were lysed, and total cell lysates were subjected to Western blot analysis. The same blots were reprobed with β-tubulin Ab to ensure equal loading. Representative data from three experiments are shown.

To assess the signaling function of both human CD72fl and the CD72Δex8 isoforms, we constructed retroviral vectors containing FLAG-tagged CD72fl, CD72Δex8 isoform, or their YF mutants in which the tyrosine residue at ITIM was replaced by phenylalanine. Although BCR ligation by antigen but not anti-Ig antibody induces efficient mouse CD72-mediated BCR regulation through recruitment of SHP-1, human B cell lines including Raji and Ramos exhibited reduced SHP-1 expression [13], and expressed endogenous CD72 and BCR with unknown antigen specificity [14]. We therefore transduced these retroviral vectors into the mouse B cell line K46μv, which we used to examine the signaling function of mouse CD72 because of its expression of BCR reactive to the hapten (4-hydroxy-3-nitrophenyl) acetyl (NP) without endogenous CD72 expression [9]. All the K46μv transfectants expressed similar levels of NP-reactive BCR (Figure 2A), and BCR ligation by a specific antigen NP-BSA induced phosphorylation of extracellular signal-regulated kinase (ERK) and Ca^2+^ influx (Figure 2B, C). However, both ERK phosphorylation and Ca^2+^ influx induced by antigen stimulation were decreased in K46μv CD72fl transfectants compared to K46μv cells transduced with vector alone (K46μv-vector) (Figure 2B, C), indicating that CD72fl negatively regulates BCR signaling. In contrast, neither ERK phosphorylation nor Ca^2+^ flux was decreased in K46μv transfectants that express the YF mutant of CD72fl (CD72flYF) (Figure 2B, C), indicating that CD72fl-induced BCR regulation depends on its ITIM. Accordingly, CD72fl but not CD72flYF was markedly phosphorylated and associated with SHP-1 after antigen stimulation (Figure 2D). Human CD72fl thus negatively regulates BCR signaling in mouse B cells by recruiting SHP-1 at the phosphorylated ITIM, as is the case for mouse CD72 [4-6].

In K46μv transfectants, surface expression of CD72Δex8 isoform was much lower than that of CD72fl (Figure 2A), although the amount of CD72Δex8 in total cell lysates was much larger than that of CD72fl (Figure 2E), suggesting poor efficiency of transport of CD72Δex8 to the cell surface. Equivalent ERK phosphorylation and Ca^2+^ influx by NP-BSA treatment in K46μv CD72Δex8 cells indicate that CD72Δex8 does not regulate BCR signaling. CD72Δex8 is thus poorly expressed on the cell surface and does not regulate BCR signaling.

### Increased CD72Δex8 expression in CD72*2-carrying peripheral B-cells

**Figure 3:**
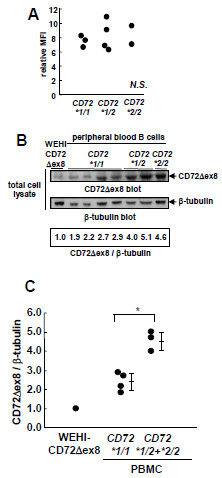
Protein level of endogenous CD72Δex8 is increased in peripheral blood B cells in individuals with CD72*2. A: Levels of cell surface expression of CD72 in individuals with each *CD72* genotype. Peripheral blood B cells were isolated from 9 healthy donors (three CD72*1/1, four CD72*1/2, and two CD72*2/2). Cell surface expression was analyzed by flow cytometry using a FACSCalibur. **B** and **C**: Expression of CD72Δex8 in B cells from individuals of each *CD72* genotype and WEHI-CD72Δex8 transfectants. Peripheral blood B cells were isolated from 7 healthy donors (four CD72*1/1, two CD72*1/2 and one CD72*2/2) by autoMACS. Cells were lysed, and total cell lysates were subjected to Western blot analysis using antibody specific to CD72Δex8 (**B**). The same blots were reprobed with anti-β-tubulin Ab to ensure equal loading. Levels of expression of CD72Δex8 were standardized to that of β-tubulin. Data are shown in dot plots (**C**). CD72Δex8 levels in peripheral blood B cells correlate to the number of CD72*2 (*P* < 0.05; Mann–Whitney U test). Values are the mean ± SE. Representative data from three experiments are shown.

Since the ratio of CD72Δex8 mRNA to CD72fl mRNA is increased in B cells from CD72*2-carrying individuals resistant to SLE [7], we next determined the protein levels of endogenous CD72fl and CD72Δex8 in human primary B cells. When we examined the surface expression of CD72 by flow cytometry using an anti-CD72 antibody that reacts to both CD72fl and CD72Δex8, peripheral blood B cells from healthy individuals carrying CD72*1/1, CD72*1/2, or CD72*2/2 exhibited similar levels of surface CD72 expression (Figure 3A). This finding suggests that expression of CD72fl does not differ among various *CD72* haplotypes because CD72Δex8 is only poorly expressed on the surface. To examine the CD72Δex8 level, we generated polyclonal antibodies that recognize CD72Δex8 but not CD72fl, and measured the protein levels of CD72Δex8 corrected for the level of β-tubulin. The CD72Δex8 levels in peripheral blood B cells carrying CD72*2 including both CD72*1/2 and CD72*2/2 were significantly higher than those carrying CD72*1/1 (Figure 3B, C; CD72*1/1 vs. CD72*1/2 + *2/2, Mann–Whitney U test: *P* < 0.05). Taken together, these findings indicate that B cells carrying CD72*2, a haplotype protective against SLE, express higher levels of CD72Δex8 whereas levels of CD72fl were similar regardless of the number of CD72*2. Increase in the CD72Δex8 level is thus responsible for CD72*2-mediated resistance to SLE, although CD72Δex8 does not regulate BCR signaling.

### CD72Δex8 accumulates in ER

**Figure 4:**
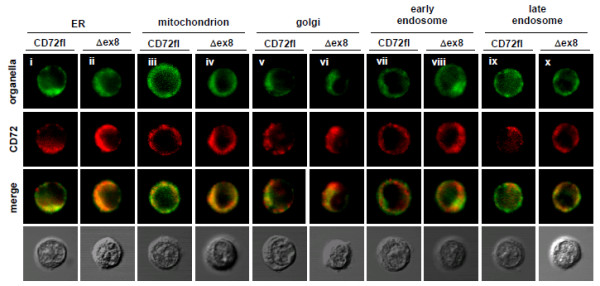
**Localization of CD72Δex8 in WEHI-231 cells.** Intracellular localization of CD72fl and CD72Δex8 in WEHI-231 transfectants. Because of the relatively low fluorescence of CD72fl, its fluorescence intensity was amplified. CD72fl (i, iii, v, vii, ix) and CD72Δex8 (ii, iv, vi, viii, x) are shown in red. Proteins localized in ER (i,ii), mitochondria (iii,iv), Golgi apparatus (v,vi), early endosomes (vii,viii), and late endosomes (ix,x) are shown in green. Cells were observed by confocal microscopy. Representative data are shown.

**Figure 5:**
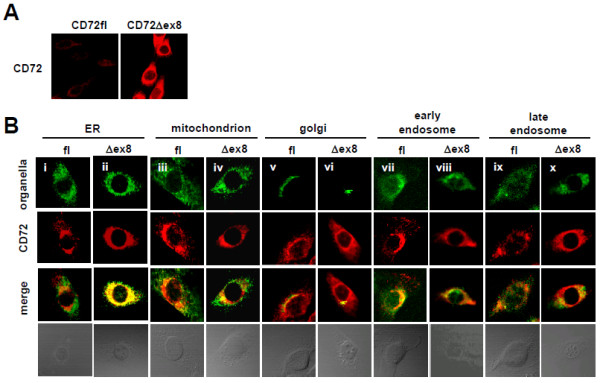
**CD72Δex8 is accumulated in ER. A**: Intracellular staining CD72 in Balb/c-3T3 transfectants. **B**: Intracellular localization of CD72fl and CD72Δex8 in Balb/c-3T3 transfectants. Because of the relatively low fluorescence of CD72fl, its fluorescence intensity was amplified. Proteins localized in ER (i, ii), mitochondria (iii, iv), Golgi apparatus (v, vi), early endosomes (vii, viii), and late endosomes (ix, x) are shown in green. CD72fl and CD72Δex8 are shown in red. Cells were observed by confocal microscopy. Representative data from five experiments are shown.

Since CD72Δex8 is poorly expressed on the cell surface, we next examined the intracellular localization of CD72fl and CD72Δex8 by confocal microscopy. In mouse B-cell line WEHI-231 transfectants, CD72Δex8 was accumulated inside the cells, though its location was unclear due to the relatively small cytoplasm (Figure 4). To determine intracellular localization in detail, we then transduced retroviral vectors encoding CD72fl and CD72Δex8 into Balb/c-3T3 cells, which contain much larger cytoplasm than B lymphocytes. Fluorescence for CD72 was much stronger in CD72Δex8 transfectants than in CD72fl transfectants, indicating that CD72Δex8 is accumulated inside the cells (Figure 5A). We therefore amplified the fluorescence intensity of CD72fl to the levels of CD72Δex8, and compared the intracellular localization of these proteins. Both CD72fl and CD72Δex8 were localized in ER, mitochondria, Golgi apparatus, early endosomes, and late endosomes, which are reasonable locations for membrane proteins (Figure 5B). However, CD72Δex8 was strongly accumulated in ER compared to CD72fl (Figure 5B).

**Figure 6:**
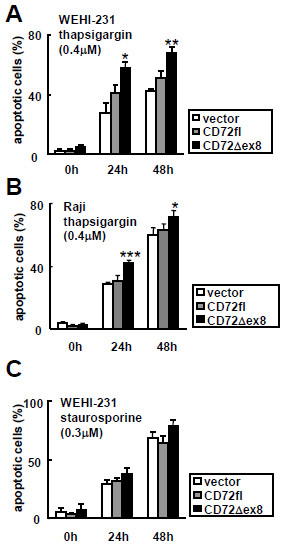
**CD72Δex8 augments and ER stress-induced apoptosis in the mouse B cell line WEHI-231 and human B cell line Raji. A** and **B**: Enhancement of apoptosis by CD72Δex8 in WEHI-231 (**A**) and Raji (**B**). **C**: cells were stimulated with staurosporine. Apoptotic cells containing hypodiploid DNA were analyzed by flow cytometry using a FACSCalibur. Values are the mean ± SD of triplicate determinations. *P < 0.01, **P < 0.001, ***P < 0.0001 (Student’s t-test). Representative data from three experiments are shown.

Eukaryotic cells react rapidly to dysfunction of the ER through a set of evolutionarily conserved adaptive pathways known as the unfolded protein response (UPR), and persistent or intense ER stress triggers apoptosis [15,16]. To obtain additional evidence for ER localization of CD72Δex8, we addressed whether CD72Δex8 augments ER stress-mediated apoptosis. Expression of FLAG-tagged CD72Δex8 but not CD72fl enhanced apoptosis of mouse B cell line WEHI-231 and human B cell line Raji induced by thapsigargin, which generates ER stress by depletion of Ca^2+^ from ER [17] (Figure 6A, B). In contrast, CD72Δex8 did not augment apoptosis of WEHI-231 transfectants induced by treatment with staurosporine, which induces apoptosis by mitochondrial stress [18] (Figure 6C). Thus, CD72Δex8 specifically enhances ER stress-induced apoptosis.

## Discussion

Here we addressed humoral immunity and expression of the two isoforms of human CD72, i.e., CD72fl and CD72Δex8, in healthy individuals carrying different *CD72* haplotypes as the coding sequences of these isoforms were the same regardless of different *CD72* haplotypes. Individuals carrying higher numbers of CD72*2 allele showed lower serum levels of IgG, clearly demonstrating association of, CD72*2 with low serum Ig as well as reduced risk for SLE [7]. The cell surface expression level of CD72fl on peripheral B cells was similar regardless of the number of CD72*2 allele. This is in agreement with the previous study in which flow cytometry demonstrated relatively homogeneous CD72 expression on B cell surface in healthy individuals [19]. Thus, BCR signal regulation mediated by CD72fl in B cells does not differ among each *CD72* genotypes because BCR regulation appears to occur on the cell surface. In contrast, Individuals carrying higher numbers of CD72*2 allele showed higher levels of CD72Δex8 in peripheral B cells. Although the sample size of this analysis is relatively small, this result is in agreement with our previous study with a larger sample size (n=32) demonstrating that the ratio of CD72Δex8 to CD72fl mRNA correlates to the number of CD72*2 allele [7], and suggests that presence of CD72*2 allele increases the protein level of CD72Δex8 but not CD72fl. Taken together, CD72Δex8 but not CD72fl is responsible for difference in serum Ig and risk for SLE among individuals carrying different *CD72* genotypes.

We further examined the functional activity of CD72fl and CD72Δex8, in signal regulation and cellular localization by expressing these isoforms in mouse B cell lines K46μv and WEHI-231, human B cell line Raji and fibroblasts. As is the case for mouse CD72, CD72fl negatively regulated BCR signaling in ITIM-dependent fashion. In contrast, CD72Δex8 did not regulate BCR signaling, probably due to its poor surface expression. CD72Δex8 was accumulated in ER in fibroblasts and augmented apoptosis induced by ER-stress in WEHI-231 and Raji, demonstrating ER localization of CD72Δex8. Thus, ER-localizing CD72Δex8 is responsible for the regulation of antibody production as well as susceptibility to SLE mediated by *CD72* polymorphism.

CD72Δex8 is generated by alternative splicing that skips exon 8, causing replacement of an exon 8-encoded stretch of 42 amino acids in the C-type lectin domain in the extracellular region by a totally different sequence of 49 amino acids encoded by exon 9 [7]. This extensive change in the extracellular C-type lectin domain may cause accumulation of CD72Δex8 in ER. The change in the C-type lectin domain may induce misfolding of the CD72Δex8 protein, which causes ER retention of the protein. Splicing isoforms of surface proteins such as human FcγRIb, mouse glutamate receptor 7b (GluR7b), and rat voltage-dependent and Ca2^+^−activated K^+^ channel (MaxiK) are mostly retained in the ER [20-22]. Whether these ER-localized isoforms play a role in cellular functions need to be elucidated in future studies.

We previously demonstrated that CD72*2 specifically abrogates the susceptibility to SLE conferred by the allele of FcγRIIb carrying the substitution of 232Ile by 232Thr (*FCGR2B-Ile232Thr*) [7], which is suggested to reduce the inhibitory function of FcγRIIb [23]. FcγRIIb-deficient mice exhibit loss of B cell self-tolerance at the IgG^+^ B cell and plasma cell stages, and development of SLE-like disease [24], demonstrating that FcγRIIb provides a distal peripheral checkpoint limiting the accumulation of self-reactive plasma cells and thereby preventing SLE. The Ile232Thr substitution may thus reduce the activity of FcγRIIb in maintaining self-tolerance, and thereby increase risk for SLE. Our findings suggest that CD72*2 augments apoptosis of B cells by enhancing expression of CD72Δex8 (Figure 6). Augmented B cell apoptosis may reduce the number of self-reactive B cells, and autoantibody-producing plasma cells may not be generated at high frequency in CD72*2-carrying individuals even in the presence of inefficient self-tolerance caused by *FCGR2B-Ile232Thr*. Recently, over 30 loci were reported as the genes significantly associated with susceptibility to SLE by genome-wide association study (GWAS) [25]. However, most of these loci carry very small effect, and the proportion of heritability explained by these variants is modest [26,27]. Other than the effect of causal rare variant, it is possible to explain a part of “missing heritability” by the gene-gene interaction. This study, which shows the functional interaction between *CD72* and *FCGR2B*, will be a functional evidence of such a gene-gene interaction.

Increasing numbers of studies have identified disease-susceptibility alleles associated with specific splicing patterns [28]. Our previous [7] and the present studies have demonstrated that the genetic polymorphism of human *CD72* associated with susceptibility to SLE regulates the efficiency of alternative splicing, although how the polymorphism affects alternative splicing is not known. Further elucidation of the molecular mechanisms of splice variations and their functional significance will provide important clues to the pathogenesis of autoimmune diseases and development of new therapeutic approaches.

## Conclusion

Our finding that serum IgG levels in individuals carrying the CD72*2 allele including both individuals with CD72*1/2 and those with CD72*2/2 are significantly lower than those in individuals carrying CD72*1/1 clearly demonstrates that human *CD72* polymorphism regulates humoral immunity as well as risk for SLE [7]. Of the two splicing isoforms of human CD72, CD72Δex8 appears to be responsible for the regulation of Ig production and autoimmunity, as the expression level of CD72Δex8 but not the other isoform CD72fl is different between B cells from individuals with CD72*2 and those with CD72*1/1. Because CD72Δex8 is accumulated in ER and poorly transported to the cell surface, CD72Δex8 may regulate B cell activity by a mechanism distinct from CD72fl that is expressed on the surface and regulate signaling through BCR as is the case for mouse CD72. Although overexpression of CD72Δex8 enhances ER stress-induced apoptosis, further studies are required to elucidate how CD72Δex8 in ER regulates B cell activity.

## Abbreviations

BCR: B cell antigen receptor; ER: Endoplasmic reticulum; ERK: Extracellular signal-regulated kinase; GWAS: Genome-wide association study; Ig: Immunoglobulin; ITIM: Immunoreceptor tyrosine-based inhibition motif; NP: Hapten (4-hydroxy-3-nitrophenyl) acetyl; SHP-1: Src homology 2 domain-containing protein tyrosine phosphatase-1; SLE: Systemic lupus erythematosus.

## Competing interest

The authors declare that they have no competing interests.

## Authors’ contributions

Study design: YH, TA, NT, ZH, KT, TT; data analysis: YH, TA, TT; manuscript writing: YH, NT, KT, TT; patient recruitment: NT; collecting data: YH, TA, NT. All authors read and approved the final manuscript.
